# The effectiveness and safety of extracorporeal shock wave lithotripsy for the management of kidney stones: A protocol of systematic review and meta-analysis

**DOI:** 10.1097/MD.0000000000019915

**Published:** 2020-05-08

**Authors:** Yong-chun Qiang, Yu-ge Guo, Yun-qi Wang

**Affiliations:** a Department of Urology, Xianyang Hospital of Yan’an University; b Department of Obstetrics and Gynecology; c Department of Urology, Yangling Demonstration District Hospital, Xianyang, China.

**Keywords:** effectiveness, extracorporeal shock wave lithotripsy, kidney stones, safety

## Abstract

**Background:**

This study will assess the effectiveness and safety of extracorporeal shock wave lithotripsy (ESWL) for patients with kidney stones (KS).

**Methods:**

A comprehensive and systematic literature records search for studies will be conducted in MEDLINE, EMBASE, Cochrane Library, WANGFANG, VIP, Chinese Biomedical Literature Database, and China National Knowledge Infrastructure. All these databases will be searched from inception to the present without language limitation. Cochrane risk of bias tool will be used to assess the methodological quality for all included studies. Statistical analysis is performed using RevMan 5.3 software.

**Results:**

This study will provide synthesis of current evidence of ESWL for patients with KS through assessing primary outcomes of overall stone-free rate, and secondary outcomes of mean stone size (mm), pain intensity, urinary biochemical variables, mean hospital stay (day), quality of life, and adverse events.

**Conclusion:**

This study will provide recommendations for the effectiveness and safety of ESWL for patients with KS, which may help to guide clinician.

**PROSPERO registration number:**

PROSPERO CRD42019157243.

## 1 Introduction

Kidney stones (KS), also known as nephrolithiasis, is a very common urological disease.^[1–4]^ It has been estimated that its prevalence rates are up to 14.8% and increasing, and its recurrence rates are up to 50% within the subsequent 5 to 10 years after the first episode.^[5,6]^ If it cannot be treated effectively, it can cause significant morbidity, and can seriously impact quality of life in patients with KS.^[7–9]^ Risk factors including obesity, diabetes mellitus, hypertension and metabolic syndrome contribute to the KS formation.^[10–13]^ A variety of managements for KS are available, such as acupuncture, herbal medicine, surgery, dietary supplementation, oral medicine, and extracorporeal shock wave lithotripsy (ESWL).^[14–21]^ A numerous studies have reported that ESWL can effectively treat patients with KS.^[22–32]^ Although a previous related systematic review has been conducted in 2014,^[24]^ there still several high quality clinical trials have been published after that.^[25–32]^ Therefore, this update study will investigate the effectiveness and safety of ESWL for the treatment of KS.

## 2 Methods

### 2.1 Study registration

We have registered this study on PROSPERO (CRD42019157243). It has been reported following the guidelines of Preferred Reporting Items for Systematic Review and Meta-Analysis Protocols Statement.^[33]^

### 2.2 Eligibility criteria

#### 2.2.1 Type of studies

We will include randomized controlled trials (RCTs) that focused on the ESWL for patients with KS without language and publication status limitations. However, non-RCTs, and quasi-RCTs will not be included.

#### 2.2.2 Type of participants

All included participants must be diagnosed with KS, regardless of country, ethnic background, gender, age, and economic status.

#### 2.2.3 Type of interventions

Any forms of ESWL intervention alone has been assigned to the patients in the experimental group.

The intervention in the control group could be any management, except the ESWL.

#### 2.2.4 Type of outcomes

The primary outcomes is overall stone-free rate. The secondary outcomes are mean stone size (mm), pain intensity, urinary biochemical variables, mean hospital stay (day), quality of life, and adverse events.

### 2.3 Search strategy

We will search the following electronic bibliographic databases comprehensively and systematically from their inception to the present: MEDLINE, EMBASE, Cochrane Library, WANGFANG, VIP, Chinese Biomedical Literature Database, and China National Knowledge Infrastructure. All databases will be searched without language limitation. The search keywords include kidney stones, renal lithiasis, nephrolithiasis, extracorporeal shock wave lithotripsy, ESWL, shock waves, random, randomly, controlled trial, clinical trial, blind, control, comparator, allocation, and concealment. The detailed search strategy for MEDLINE is demonstrated in Table 1. Similar search strategies will be built for other electronic databases.

**Table 1:**
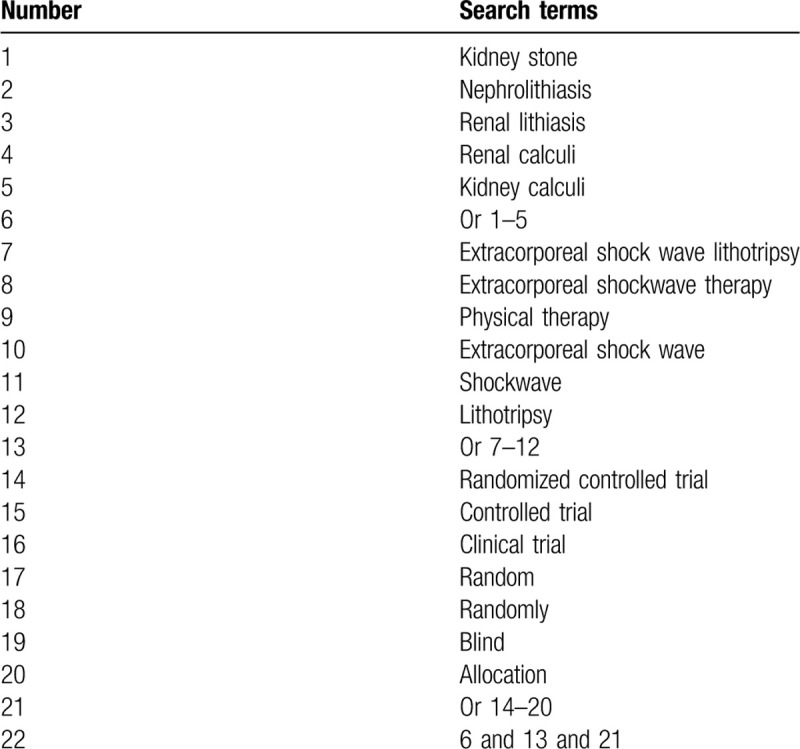
Search strategy for MEDLINE.

Additionally, we will also search gray literature sources, such as conference proceedings, dissertations, clinical trial registry, and reference lists of included studies.

### 2.4 Data selection and extraction

#### 2.4.1 Study selection

**Figure 1:**
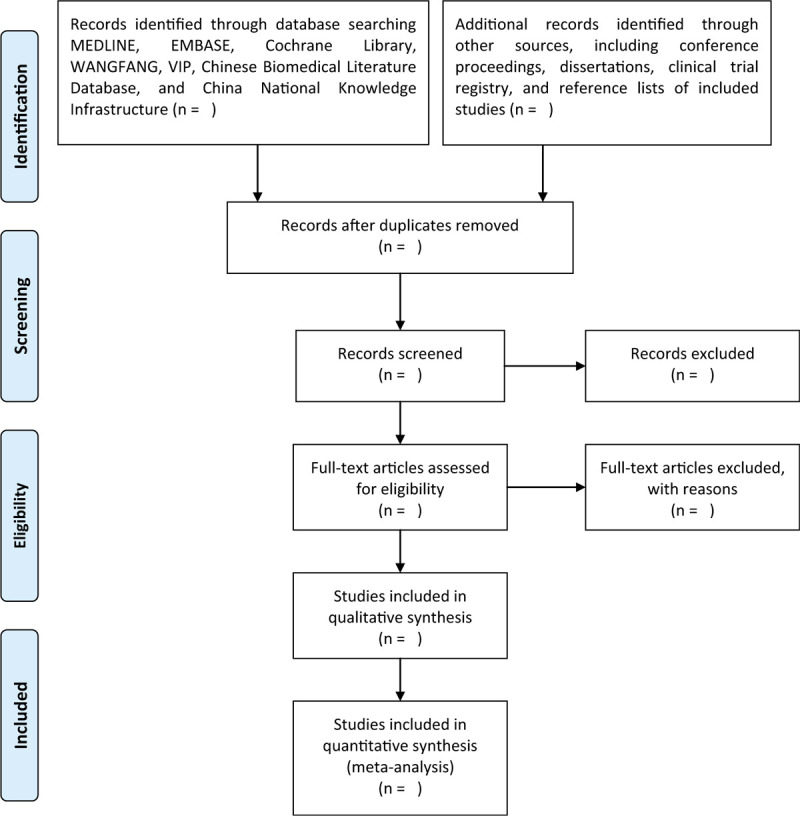
Flowchart of study selection.

Two researchers are independently responsible for the study selection based on the previously defined study inclusion criteria. All retrieved studies will be scanned in the forms of titles and abstracts initially, and all unqualified and duplicated studies will be removed. Then, we will read full texts of the remaining studies for further selection. All excluded literatures will be recorded separately with detailed reasons. In case of any different opinions between two researchers, a third researcher will help to make decision through negotiation. The process of study selection will be presented in the flowchart in Figure 1.

#### 2.4.2 Data extraction

The following information will be independently extracted by two researchers through pre-designed standard sheet: first author, publication year, location, race, age, sex, disease duration and duration, diagnostic criteria, eligibility criteria, sample size, study setting, methods of randomization, blind, concealment, treatment details, all outcome measurements, safety, and funding information. We will contact primary authors by email if essential information is missing or unclear. A third researcher will help to solve any disagreements between two researchers by discussion if necessary.

### 2.5 Study quality assessment

Two researchers will evaluate the risk of bias for all included RCTs using Cochrane Risk of Bias Tool. We will assess each study at 7 levels, and each one is divided into 3 degrees: low, unclear, and high risk of bias. The differences between two researchers will be solved by consensus via discussion.

### 2.6 Data analysis

RevMan 5.3 software is utilized for statistical analysis. All continuous data will be calculated as mean difference or standardized mean difference and 95% confidence intervals (CIs), while all dichotomous will be calculated as risk ratio and 95% CIs. Statistical heterogeneity across the eligible studies will be checked by *I*^2^ statistic. *I*^2^ ≤ 50% indicates low level of heterogeneity, and we will choose a fixed-effects model. *I*^2^ > 50% means obvious level of heterogeneity, and we will select a random-effects model. When the heterogeneity of the merged outcome results across studies is low, we will plan to perform a meta-analysis if more than two RCTs are similar in study and patient characteristics, interventions, controls, and outcomes. In contrast, we will perform a subgroup analysis to check possible reasons for the obvious heterogeneity. We will not conduct a meta-analysis if obvious heterogeneity still be checked after subgroup analysis. Instead, we will report outcome results as a narrative summary.

### 2.7 Subgroup analysis

When there is obvious heterogeneity among included studies, we will perform a subgroup analysis in accordance with different study qualities, treatments, controls, and outcome measurements.

### 2.8 Sensitivity analysis

We will also carry out a sensitivity analysis to check the robustness of merged outcome results by removing low quality studies.

### 2.9 Reporting bias

When there are at least 10 included RCTs, we will conduct Funnel plot^[34]^ and Egger's regression test^[35]^ to identify any possible reporting bias.

### 2.10 Ethics and dissemination

No individual patient data will be involved in this study, thus, no ethic approval is needed. We will publish this study at a peer-reviewed journal.

## 3 Discussion

A numerous studies have reported that patients with KS can achieve encouraging benefits after ESWL treatment. However, their results are still not consistent. Although a recent associated systematic review has been published,^[24]^ there is still several high quality RCTs address this issue after that.^[25–32]^ Therefore, the purpose of this study is to update and to determine the effectiveness and safety of ESWL for patients with KS. This study may still have two limitations. First, some trials may have small sample size, which may affect results of this study. Second, the overall quality of some studies may be still low, which may impact study findings.

## Author contributions

**Conceptualization:** Yong-chun Qiang, Yu-ge Guo, Yun-qi Wang.

**Data curation:** Yong-chun Qiang, Yu-ge Guo, Yun-qi Wang.

**Formal analysis:** Yong-chun Qiang.

**Investigation:** Yun-qi Wang.

**Methodology:** Yu-ge Guo.

**Project administration:** Yun-qi Wang.

**Resources:** Yong-chun Qiang, Yu-ge Guo.

**Software:** Yong-chun Qiang, Yu-ge Guo.

**Supervision:** Yun-qi Wang.

**Validation:** Yong-chun Qiang, Yu-ge Guo, Yun-qi Wang.

**Visualization:** Yong-chun Qiang, Yu-ge Guo, Yun-qi Wang.

**Writing – original draft:** Yong-chun Qiang, Yun-qi Wang.

**Writing – review & editing:** Yong-chun Qiang, Yu-ge Guo, Yun-qi Wang.
